# Size-Dependent Melting Behavior of Colloidal In, Sn, and Bi Nanocrystals

**DOI:** 10.1038/srep16353

**Published:** 2015-11-17

**Authors:** Minglu Liu, Robert Y. Wang

**Affiliations:** 1 Mechanical Engineering, Arizona State University, Tempe, Arizona 85287; 2 Materials Science & Engineering, Arizona State University, Tempe, Arizona 85287; 3 Chemical Engineering, Arizona State University, Tempe, Arizona 85287.

## Abstract

Colloidal nanocrystals are a technologically important class of nanostructures whose phase change properties have been largely unexplored. Here we report on the melting behavior of In, Sn, and Bi nanocrystals dispersed in a polymer matrix. This polymer matrix prevents the nanocrystals from coalescing with one another and enables previously unaccessed observations on the melting behavior of colloidal nanocrystals. We measure the melting temperature, melting enthalpy, and melting entropy of colloidal nanocrystals with diameters of approximately 10 to 20 nm. All of these properties decrease as nanocrystal size decreases, although the depression rate for melting temperature is comparatively slower than that of melting enthalpy and melting entropy. We also observe an elevated melting temperature during the initial melt-freeze cycle that we attribute to surface stabilization from the organic ligands on the nanocrystal surface. Broad endothermic melting valleys and very large supercoolings in our calorimetry data suggest that colloidal nanocrystals exhibit a significant amount of surface pre-melting and low heterogeneous nucleation probabilities during freezing.

Colloidal nanocrystals are made via solution-phase chemistry and consist of an inorganic core with organic ligands bound to its surface. Rapid progress in the field of colloidal nanocrystal synthesis^1^,^2^ has led to their use in numerous applications such as LEDs^3^,^4^, optoelectronics^5^,^6^, electronics^7^,^8^, thermal storage^9^,^10^, and thermoelectrics^11^,^12^. One potential concern for using colloidal nanocrystals in these applications is that their lower melting temperatures may be incompatible with elevated temperatures during device operation and/or fabrication. It is well known that the melting temperature of nanoparticles decreases as their characteristic size decreases and this phenomenon is commonly referred to as melting point depression^13^,^14^,^15^,^16^,^17^,^18^,^19^,^20^,^21^,^22^. However, the vast majority of melting point depression studies focus on a sub-monolayer of nanoparticles prepared by dewetting of a thin film. Nanoparticles prepared via other methods such as ball milling and/or colloidal synthesis are more commonly used and could exhibit different behavior due to variations in structure-property-processing relations. In addition, nanoparticles are often embedded in matrices, which remove the free surface of dewetted nanoparticles and also change melting behavior.

In this study, we focus on the melting behavior of colloidal nanocrystals, whose phase change properties have been largely unexplored. Goldstein *et al.*^16^ used transmission electron microscopy (TEM) to measure colloidal nanocrystal melting temperature, but did not measure melting enthalpy. Calorimetry measurements can determine both melting temperature and melting enthalpy, and a few other researchers have used this approach to investigate colloidal nanocrystal melting^23^,^24^,^25^,^26^. Unfortunately, extracting size-dependent melting behavior from these experiments is problematic because the colloidal nanocrystals were poorly isolated from one another^24^,^26^. This led to nanocrystal coalescence during the calorimetry measurements and manifested itself as a melting temperature that drifted toward bulk values during thermal cycling^25^. In some cases, the nanoscale dimensions of the nanocrystals degraded so fast that bulk melting temperatures were observed during the first melting cycle^23^,^24^.

We demonstrate that this nanocrystal coalescence problem can be completely mitigated by isolating the colloidal nanocrystals from one another via dispersion within a polymer matrix. Since colloidal nanocrystals and polymers have similar solubility and are easily mixed in a variety of solvents, this preparation of embedded nanocrystals requires little additional effort. We use polyimide resin (PI resin) for the polymer matrix because it has a glass transition temperature above 305 °C. This makes it a suitable matrix for studying the low melting temperature metals in this work. Specifically, we focus on the melting behavior of In, Sn, and Bi nanocrystals whose bulk melting temperatures are 157, 232, and 271 °C, respectively. The use of the stabilizing PI resin matrix allows us to observe highly repeatable melting behavior throughout numerous melt-freeze cycles. We show that the colloidal nanocrystal melting temperatures, melting enthalpies, and melting entropies are all size-dependent and decrease as nanocrystal diameter decreases. We also observe an elevated melting temperature during the initial melt-freeze cycle that we attribute to surface stabilization from the nanocrystal’s organic ligands. Lastly, we observe signatures of surface pre-melting and low heterogeneous nucleation probabilities in our samples that manifest themselves as broad endothermic melting valleys and very large supercooling in the calorimetry data.

## Results and Discussion

### Melting Temperatures

**Figure 1:**
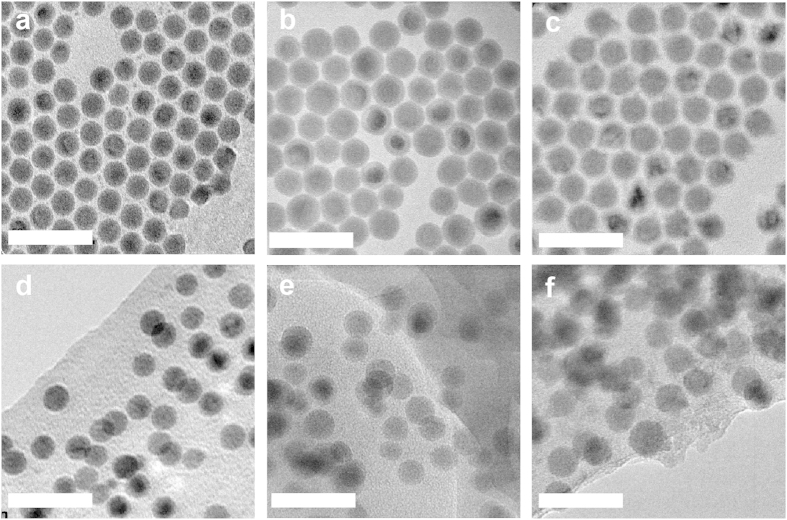
Transmission electron microscopy images of (**a**) 14.9 ± 0.7 nm Bi nanocrystals, (**b**) 17.0 ± 1.1 nm Sn nanocrystals, and (**c**) 17.0 ± 1.1 nm In nanocrystals. Corresponding images of the (**d**) Bi, (**e**) Sn, and (**f**) In nanocrystals after dispersion into the polyimide resin matrix illustrate that the size and shape of the nanocrystals are preserved during sample preparation (the scale bar is 50 nm).

We prepared our samples by synthesizing colloidal nanocrystals and then dissolving them with PI resin in a shared solvent. We then drop-cast the solution and removed the solvent *via* heating. We synthesized our colloidal nanocrystals using hot-injection techniques reported by Kravchyk *et al.*^27^ and Yarema *et al.*^28^,^29^,^30^, and controlled the nanocrystal diameter between 10 and 20 nm by varying the reaction temperature and time (Figure S1). The transmission electron microscope (TEM) images in Fig. 1a–c show the excellent shape and size uniformity of the nanocrystals prepared using this approach. The images in Fig. 1d–f illustrate that the nanocrystal size and shape are preserved when embedded in the PI resin matrix.

**Figure 2:**
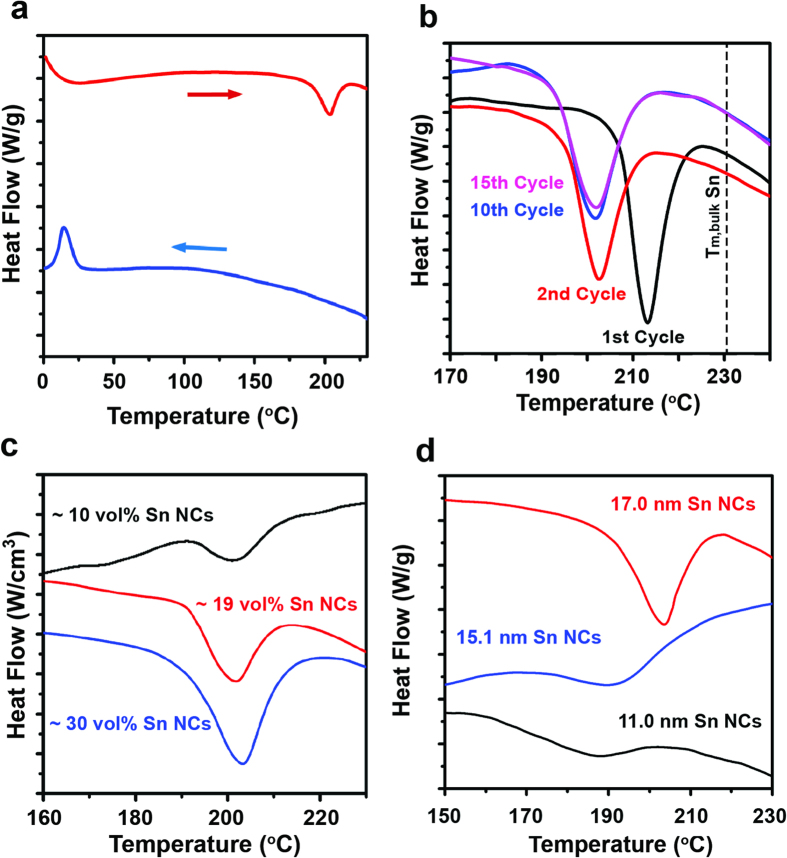
(**a**) A typical heating (red curve) and cooling (blue curve) in a melt-freeze cycle during differential scanning calorimetry (DSC) measurements. This specific sample consists of 17 nm Sn nanocrystals (NCs) dispersed in a polyimide resin matrix. Note that the feature located in the 0–25 °C range of the heating curve is a measurement artifact that occurs when the DSC switches from cooling to heating. (**b**) The endothermic valley of 17 nm Sn nanocrystals during several different melt-freeze cycles. After the initial melting cycle, a stable and repeatable melting temperature and melting enthalpy are observed. We attribute the elevated melting temperature during the initial cycle to surface stabilization from the organic ligands on the nanocrystal surface. (**c**) The endothermic valley of 17 nm Sn nanocrystals prepared at varying nanocrystal volume fractions within the polyimide resin matrix. As the nanocrystal volume fraction is changed, the melting enthalpy signature increases proportionately and the melting temperature remains unchanged. (**d**) The endothermic melting valley for Sn nanocrystals of varying diameters that are embedded in a polyimide resin matrix. As the nanocrystal diameter decreases, both the melting temperature and melting enthalpy decrease. The full-width at half-maximum of the melting valley also increases for smaller nanocrystals.

The typical melting behavior of a colloidal nanocrystal sample dispersed in a PI resin matrix is illustrated in Fig. 2a. This figure shows a DSC measurement on 17 nm Sn nanocrystals that have gone through multiple melt-freeze cycles (see Figure S2a and S2b for measurements on Bi and In nanocrystals). An endothermic valley is observed at ~200 °C during heating and an exothermic peak is observed at ~20 °C during cooling. We attribute the endothermic valley to Sn nanocrystal melting and the exothermic peak to Sn nanocrystal freezing. These assignments are corroborated with a control DSC measurement on pure PI resin, which shows no discernable features throughout the entire temperature range (Figure S3a). Note that bulk Sn melts at 232 °C and so our observed nanocrystal melting temperature is consistent with melting point depression.

We observe an elevated melting temperature during the first melt-freeze cycle and attribute this to surface chemistry effects in colloidal nanocrystals (Fig. 2b). For example, 17 nm Sn nanocrystals melt at 214 °C during the first cycle and then melt at 204 °C during subsequent cycles. It is well known that melting typically initiates at the surface of a solid, and we postulate that the strongly bound surface ligands of colloidal nanocrystals inhibit melting by stabilizing the surface. Similar surface stabilization behavior has been observed in Pb nanoparticles in Al matrices^31^ and Ag nanoparticles in Ni matrices^32^. The lower melting temperatures in subsequent cycles suggest that the ligands detach from the nanocrystal surface during the first cycle and remain detached in subsequent cycles. This increase in initial melting temperature was observed for all of our nanocrystal compositions (In, Sn, and Bi) and diameters (10–20 nm), which suggests that this is a widespread characteristic of colloidal nanocrystals.

Our use of a PI resin matrix to isolate nanocrystals from one another prevents nanocrystal coalescence and leads to a repeatable and stable melting temperature after the initial melting cycle (Fig. 2b). Prior colloidal nanocrystal calorimetry studies have been prone to nanocrystal coalescence problems due to unsuitable matrices or the absence of matrices^23^,^24^,^25^,^26^. Consequently the observed melting temperatures drifted toward the bulk melting temperature during thermal cycling. In some cases, bulk melting temperatures could be observed during the first melting cycle^23^,^24^. This transient behavior makes measurements of size-dependent melting in colloidal nanocrystals problematic. This problem is further compounded by the increase in melting temperature observed during the first thermal cycle as described above. In our nanocrystal-PI resin samples, we are able to observe repeatable melting temperatures during thermal cycling up to a nanocrystal volume fraction of ~30% (Fig. 2c). We designate this repeatable temperature as the melting temperature of our nanocrystal samples throughout this paper.

**Figure 3:**
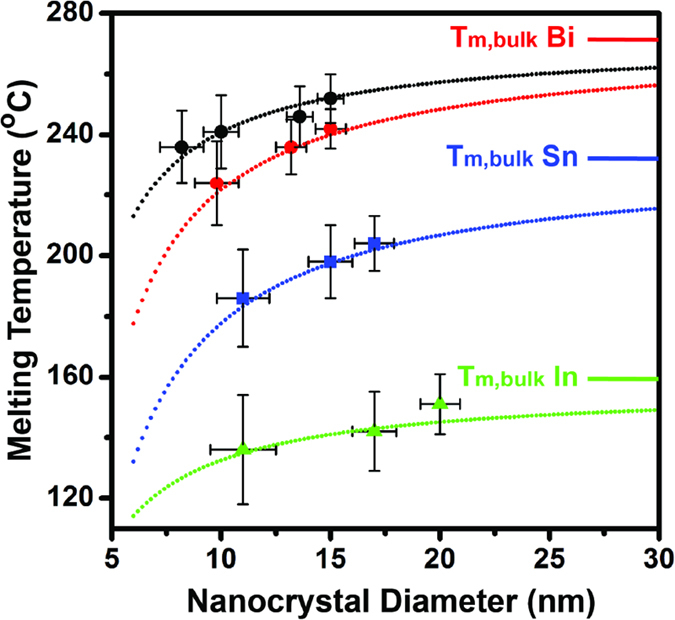
The melting temperature of colloidal In, Sn, and Bi nanocrystals as a function of nanocrystal diameter. In addition to data for In, Sn, and Bi in polyimide resin matrices (green triangles, blue squares, and red circles, respectively), this figure also contains data from our prior work on Bi nanocrystals in an Ag matrix (black circles, ref. 9). The melting temperature and melting temperature uncertainty in this figure represent the endothermic valley minimum location and endothermic valley full width at half maximum in the DSC data, respectively. The dotted curves are experimental data fits using Shi’s melting temperature model that accounts for both nanoparticle size effects and matrix effects [ref. 33].

The melting temperature of colloidal Sn, In, and Bi nanocrystals all decrease as nanocrystal diameter decreases (Fig. 3). In addition to melting temperature data collected with PI resin matrices, this figure also includes melting temperature data from our prior work on Bi nanocrystals in Ag matrices^9^. The melting point depression in the Ag matrix is notably weaker than in the PI resin matrix and highlights the importance of both size and surrounding environment. The melting model proposed by Shi^33^ takes into account the effects of both size and matrix on melting and provides an excellent fit to our data. At the heart of Shi’s model is the Lindemann criterion^34^, which states that melting occurs when the average root mean square displacement (MSD) of the atoms in a particle exceeds a critical value. Keeping in mind that surface atoms typically have a larger MSD than interior atoms, this criterion readily explains the qualitative size dependence of melting temperature. As the nanocrystal size shrinks, the number of surface atoms increases and therefore the average MSD increases. This increase in average MSD then causes the critical root MSD (i.e. melting) to be reached at lower temperatures.

Shi’s melting temperature model^33^ expands upon the Lindemann criterion by including matrix effects on the atom MSD and fits our experimental data very well. This matrix effect is captured in Shi’s model via the parameter, *α*, which is the ratio of surface atom MSD to interior atom MSD. The presence of a matrix predominantly affects the MSD of surface atoms and leaves the MSD of interior atoms largely unchanged. Shi’s model predicts a size dependent melting temperature, *T*_*m*_(*r*), that depends upon particle radius, *r*, and *α*:


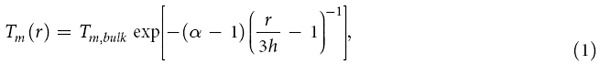


In this expression, *h* is a characteristic length representing the height of an atomic monolayer on a bulk surface and is estimated from the crystal lattice constant. We obtained reasonable fits to our experimental data on both Sn and In nanocrystals in PI resin with an *α* value of 1.54. It is intuitive that the same *α* value can be used to fit both sets of data because their matrices are the same and because Sn and In have the same crystal structure. The *α* value for Bi nanocrystals in PI resin was 1.42 and differed slightly from Sn and In. We speculate that this slight difference may arise from a difference in crystal structure, and hence a change in the value for *h* in Equation 1. The *α* value for Bi nanocrystals in an Ag matrix was 1.2 and indicates that the rigid Ag matrix suppresses MSD comparatively more than the soft PI resin matrix.

### Melting Enthalpies and Entropies

While reports on size-dependent melting temperature are widespread, reports on size-dependent melting enthalpy are relatively limited^17^,^19^,^35^. One of the primary challenges in measuring size-dependent melting enthalpy stems from the preparation of the experimental sample. Samples for size-dependent melting studies are typically prepared by vapor depositing a thin film on a substrate that subsequently dewets and forms a sub-monolayer of nanoparticles. These samples produce small thermal energy signals during phase change and require sophisticated microfabricated nanocalorimeters to determine their melting enthalpy^17^,^19^,^20^.

Our sample preparation embeds a large ensemble of nanoparticles into the PI resin matrix and therefore produces a large thermal signal that can be easily detected using standard calorimeters. However, measuring the mass of our nanoparticles requires additional care due to the structure of colloidal nanocrystals. A direct measurement of colloidal nanocrystal mass would contain mass contributions from both the nanoparticle (i.e. the nanocrystal core) and the surface ligands. To circumvent this problem, we used cyclic DSC measurements to determine the mass ratio of the nanocrystal cores to the nanocrystal ligands. Let us consider the case of colloidal Sn nanocrystals for illustrative purposes. We first prepared a DSC sample consisting of colloidal Sn nanocrystals without a PI resin matrix. We then subjected this sample to multiple melt-freeze cycles, during which the absence of a protective matrix leads to nanocrystal coalescence into a bulk material. Upon observing the bulk melting temperature of Sn (Figure S3b), we determined the true mass of Sn in our colloidal nanocrystals by comparing our measured melting enthalpy to the bulk Sn melting enthalpy (59 J/g). We did this measurement for all nanocrystal compositions and sizes because the core:ligand mass ratio depends on both of these parameters.

**Figure 4:**
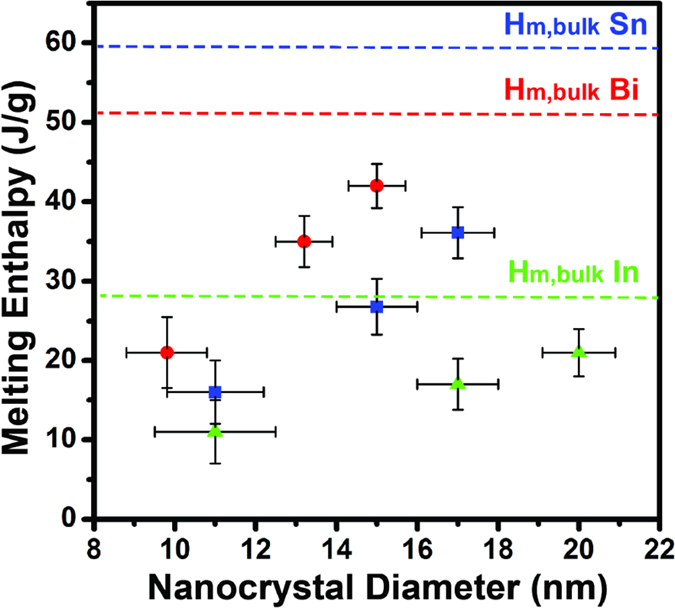
The melting enthalpy of colloidal In (green triangles), Sn (blue squares), and Bi (red circles) as a function of nanocrystal diameter. This size-dependent melting enthalpy is a consequence of size-dependence in both melting temperature and melting entropy.

The melting enthalpy of colloidal Sn, In, and Bi nanocrystals decrease away from their respective bulk values as nanocrystal size is decreased (Fig. 4). Melting enthalpy, *H*_*m*_, can be expressed as the product of melting temperature, *T*_*m*_, and melting entropy, *S*_*m*_:


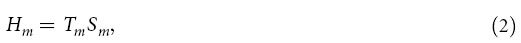


**Figure 5:**
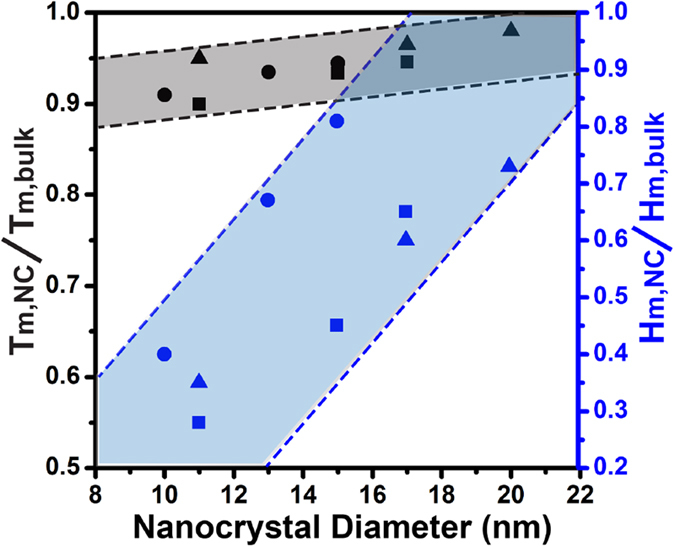
The normalized depression in melting temperature (black) and melting enthalpy (blue) as a function of nanocrystal diameter for In (triangles), Sn (squares), and Bi (circles) nanocrystals (NCs). The depression rate for melting enthalpy is significantly faster than the depression rate for melting temperature.

Since melting temperature decreases as nanocrystal diameter decreases, a corresponding depression in melting enthalpy is also expected. However, we find that melting entropy also plays a key role as indicated by the fact that the depression rate of melting enthalpy is much faster than that of melting temperature. This trend is revealed in a plot of normalized melting enthalpy depressions, H_m,NC_/H_m,bulk_, and normalized melting point depressions, T_m,NC_/T_m,bulk_, versus the nanocrystal size (Fig. 5). For example, nanocrystals with ~10 nm diameters have melting temperatures and melting enthalpies that are ~90% and ~30% of their bulk values.

The significant difference in depression rates for melting temperature and melting enthalpy means that melting entropy is also size-dependent and decreases with nanocrystal size. Melting entropy represents the difference between the solid state entropy and liquid state entropy. The entropy of a solid increases as nanoparticle diameter decreases due to the growing fraction of surface atoms, which have larger MSD^36^,^37^. This brings the entropy of solid nanoparticles closer to the liquid state entropy and leads to decreasing melting entropy as nanocrystal diameter is reduced. Figure S4 illustrates the entropy size dependence for our colloidal In, Sn, and Bi nanocrystals.

### Surface Pre-Melting and Supercooling

Unlike melting in bulk materials, melting in colloidal nanocrystals occurs over a wide finite temperature range (i.e. the melting valley has a large full-width at half-maximum, FWHM). Furthermore, the FWHM increases as nanocrystal diameter decreases (Fig. 2d). For example, the FWHM of the melting valley for Sn nanocrystals changes from 17 to 30 °C as the nanocrystal diameter shrinks from 17 nm to 11 nm. We also observe similar behavior for Bi and In nanocrystals (Figure S2c and S2d). This behavior is consistent with a phenomenon known as surface pre-melting, during which a thin liquid layer forms on the solid surface at a temperature below the conventionally recognized melting temperature^17^. While this phenomenon has been observed in bulk materials^38^,^39^,^40^, it has little effect on the overall melting behavior because of the extremely small fraction of surface atoms in bulk material. In contrast, nanoparticles have a significant fraction of surface atoms that can produce a much larger surface pre-melting signature. Furthermore, surface curvature enhances surface pre-melting^22^,^41^,^42^ and is another reason that our samples produce large pre-melting signals. To capture the effect of surface pre-melting in the reporting of our size-dependent melting data (Fig. 3), we have used the endothermic valley minimum and valley FWHM for the melting temperature and melting temperature uncertainty, respectively. We also note that while our finite nanocrystal size distribution could lead to DSC signal broadening, this mechanism is insufficient to explain our experimental measurements. The FWHM of our DSC signals are 2–11 times larger than what can be expected from nanocrystal size distribution (Table S1). In addition, control experiments with varying temperature scan rates illustrate that this parameter does not affect the DSC signal broadness either (Figure S5).

Another notable feature in the melting of colloidal nanocrystals is that they exhibit a very large amount of supercooling. For example, our 17 nm Sn nanocrystals supercooled by ~180 °C, which is approximately 40% of its melting temperature (204 °C = 477 K). Classical nucleation theory explains supercooling as resulting from the energy barrier associated with the formation of a solid-liquid interface^43^. Supercooling is typically minimized because of energetically favorable heterogeneous nucleation occurs at pre-existing interfaces and/or impurity sites. A large supercooling is regarded as evidence of homogeneous nucleation, and prior experimental efforts to maximize supercooling have found an empirical upper limit of ~0.2–0.3 T_m_^44^,^45^,^46^,^47^. One exception to this empirical upper limit is a relatively recent work on Ga nanoparticles^48^. While our In and Bi nanocrystal data exhibited supercoolings of ~0.2–0.3 T_m_, our data on Sn nanocrystals (~0.4 T_m_) represents a second exception to this empirical upper limit. We believe that the very large supercoolings observed during freezing of our samples are due to a decreased probability of heterogeneous nucleation. The probability for heterogeneous nucleation in our samples is low because colloidal nanocrystals are known to be highly defect- and impurity-free. In fact, the intentional introduction of impurities (i.e. doping) has been an ongoing challenge for the colloidal nanocrystal community^49^. These large supercooling values also suggest that the amorphous polymer matrix provides a poor heterogeneous nucleation site for crystalline metal.

## Conclusion

This systematic study on colloidal nanocrystal melting behavior provides guidance on the phase stability of these materials as they are incorporated into devices that experience elevated temperatures during operation and/or fabrication. Specifically, we have reported the melting temperatures, melting enthalpies, and melting entropies of colloidal In, Sn, and Bi nanocrystals with diameters ranging from 10–20 nm. All of these properties decreases as nanocrystal size decreases, although the depression rate for melting temperature is comparatively slower than that of melting enthalpy and melting entropy. We also observed an elevated melting temperature during the initial melting of colloidal nanocrystals and we attribute this to surface stabilization from the nanocrystal’s ligands. Broad endothermic melting valleys and very large supercooling in the calorimetry data suggest a significant amount of surface pre-melting and low heterogeneous nucleation probabilities during freezing. These observations on colloidal nanocrystal melting behavior were enabled by our new calorimetry sample preparation technique that prevents nanocrystal coalescence during melt-freeze cycles.

## Methods

### Sn Nanocrystal Synthesis

Sn nanocrystals were synthesized following the procedure reported by Kravchyk *et al.*^27^ In a typical synthesis of 17 nm Sn nanocrystals, 20 g of oleylamine (OLA) was loaded into a three-neck flask and degassed at 140 °C for 2 hours. The flask was then heated to 210 °C, at which point 3 solutions were sequentially injected. The first injection was 0.5 mmol Sn[N(SiMe_3_)_2_]_2_ dissolved in 1 mL octadecane. The second injection was 3.6 mmol LiN(SiMe_3_)_2_ dissolved in 2 mL of toluene and was injected immediately after the first injection. After 10 s, 0.6 mL of 1 M lithium triethylborohydride in tetrahydrofuan (THF) was injected, and the solution immediately turned dark. The reaction temperature was then maintained for 1 hour and then quickly cooled to room temperature using an ice bath. This reaction yields Sn nanocrystals with OLA ligands. The OLA ligands were then exchanged with oleic acid (OA) to improve nanocrystal stability. Adjusting injection temperature and growth time allows control over the nanocrystal diameter. The Sn nanocrystals were isolated from the reaction mixture by precipitating in ethanol and redispersing in a nonpolar solvent. This isolation process was repeated two additional times and the nanocrystals were dispersed in THF after the final precipitation.

#### In Precursor Synthesis and In Nanocrystal Synthesis

In nanocrystals were synthesized using the procedure reported by Yarema *et al.*^29^ The first step in this synthesis is to create the In precursor, which is In[N(SiMe_3_)_2_]_3_. To synthesize In[N(SiMe_3_)_2_]_3_, 6.6 mmol of InCl_3_ and 20 mmol of LiN(SiMe_3_)_2_ were reacted in 120 mL diethyl ether at 60 °C for 24 hours^29^. The reaction mixture was filtered through a PTFE filter and then dried under vacuum to yield a pale yellow powder. The In[N(SiMe_3_)_2_]_3_ powder was then dissolved in 15 mL of pentane, filtered, and dried again under vacuum. For In nanocrystal synthesis, 20 g of hexadecylamine (HDA) was degassed at 100 °C for 2 hours and then heated to 200 °C. Once the temperature stabilized at 200 °C, a solution of 0.26 g In[N(SiMe_3_)_2_]_3_ and 0.728 g LiN(SiMe_3_)_2_ dissolved in 8 mL toluene was injected. The reaction temperature was then cooled to ~155 °C, at which point 0.1 mL of 1 M lithium triethylborohydride in THF was injected. Depending on the desired nanocrystal diameter, the reaction temperature was maintained for an additional 1–5 minutes. The reaction mixture was then cooled using a water bath. 20 mL of toluene was added to the mixture during the cooling process to prevent the solidification of HDA. This synthesis results in In nanocrystals with HDA ligands. We exchanged these HDA ligands with OA right after synthesis to promote stability of the nanocrystal solution. The In nanocrystals were isolated from the reaction mixture using precipitation techniques in a similar manner to the Sn nanocrystals.

### Bi Precursor Synthesis and Bi Nanocrystal Synthesis

Bi[N(SiMe_3_)_2_]_3_ was used as the nanocrystal precursor for Bi nanocrystals. This precursor was synthesized via a metathesis reaction between BiCl_3_ and LiN(SiMe_3_)_2_ as reported by Yarema *et al.*^28^ In a typical synthesis, 6.6 mmol BiCl_3_ and 20 mmol LiN(SiMe_3_)_2_ were dissolved in 80 mL diethyl ether and 10 mL THF. This reaction was vigorously stirred and maintained at 0 °C for 2 hours. Upon reaction completion, white precipitates of LiCl were filtered from the mixture by a PTFE filter and the resulting solution was dried under vacuum. The resulting Bi[N(SiMe_3_)_2_]_3_ powder was then dissolved in 15 mL pentane, filtered, and again dried under vacuum. In a typical Bi nanocrystal synthesis, 20 g of HDA was degassed at 100 °C for 2 hours and then heated to 130 °C. 0.1 mL lithium triethylborohydride in THF was then injected. After 15 s, a solution of co-dissolved 0.14 g Bi[N(SiMe_3_)_2_]_3_ and 0.17 g Li[N(SiMe_3_)_2_] in 2 mL toluene was injected. After 15 s, the reaction was then stopped by cooling in a water bath. 20 mL of toluene was typically added during cooling to prevent the HDA from solidifying. This synthesis results in Bi nanocrystals with HDA ligands. We exchanged these HDA ligands with OA right after synthesis to promote stability of the nanocrystal solution. Adjusting injection temperature allows control over the nanocrystal diameter. The Bi nanocrystals were isolated from the reaction mixture using precipitation techniques in a similar manner to the Sn nanocrystals.

### Nanocrystal – PI Resin Sample Preparation

The dispersion of nanocrystals was completed using a three-step approach. First, the nanocrystals were synthesized as described above. In parallel, PI resin was dissolved in THF in a separate vial by stirring for 30 minutes. Then, an appropriate amount of nanocrystal solution and PI resin were mixed and stirred for 2 hours. The samples were then made by drop-casting the combined solution onto appropriate substrates. The nanocrystal composition and size were controlled at the nanocrystal synthesis step and nanocrystal volume fraction was controlled at the mixing step.

### Transmission Electron Microscopy (TEM)

To characterize the morphology of the nanocrystal samples, TEM (FEI Tecnai F20) was used and operated at 200 kV. The nanocrystal samples were made by drop-casting a dilute nanocrystal dispersion onto a carbon film coated copper TEM grid. The diameter of the nanocrystals was determined by statistical analysis of TEM images containing ~100 nanocrystals using ImageJ. The standard deviation of the nanocrystal diameters was used for the diameter uncertainties. The TEM samples of the nanocrystals dispersed in PI resin were prepared by drop-casting dilute combined solutions onto a SiN_X_ window.

### Differential Scanning Calorimetry (DSC)

DSC measurements were carried out using a standard differential scanning calorimeter (TA Q20). To prepare samples for DSC measurements, we drop-cast the combined solution into an aluminum DSC pan, and then heated it at 50 °C and 100 °C sequentially to remove the solvent. DSC measurements were carried out by cyclic heating and cooling at a rate of 10 °C/min. The temperature cycling range was 0 to 250 °C for Sn, 0 to 300 °C for Bi, and 0 to 200 °C for In. During these measurements, a nitrogen atmosphere was maintained using a flow rate of 50 mL/min. The DSC data was analyzed using the software provided by TA instruments.

## Additional Information

**How to cite this article**: Liu, M. and Wang, R. Y. Size-Dependent Melting Behavior of Colloidal In, Sn, and Bi Nanocrystals. *Sci. Rep.* **5**, 16353; doi: 10.1038/srep16353 (2015).

## Supplementary Material

Supplementary Information
