# Supplementary material for: Size-Dependent Melting Behavior of Colloidal In, Sn, and Bi Nanocrystals
Source: Sci Rep. 2015 Nov 17;5:16353. doi: 10.1038/srep16353 (PMC4648084; doi:10.1038/srep16353)
Supplement: Supplementary Information [file srep16353-s1.pdf]

Supplementary Information

Size-Dependent Melting Behavior of Colloidal In, Sn, and Bi Nanocrystals

Minglu Liu[1] *and Robert Y. Wang*[* 1, 2, 3]

[1]Mechanical Engineering, Arizona State University, Tempe, Arizona 85287
[2]Materials Science & Engineering, Arizona State University, Tempe, Arizona 85287
[3]Chemical Engineering, Arizona State University, Tempe, Arizona 85287

E-mail: rywang@asu.edu

Additional Figures

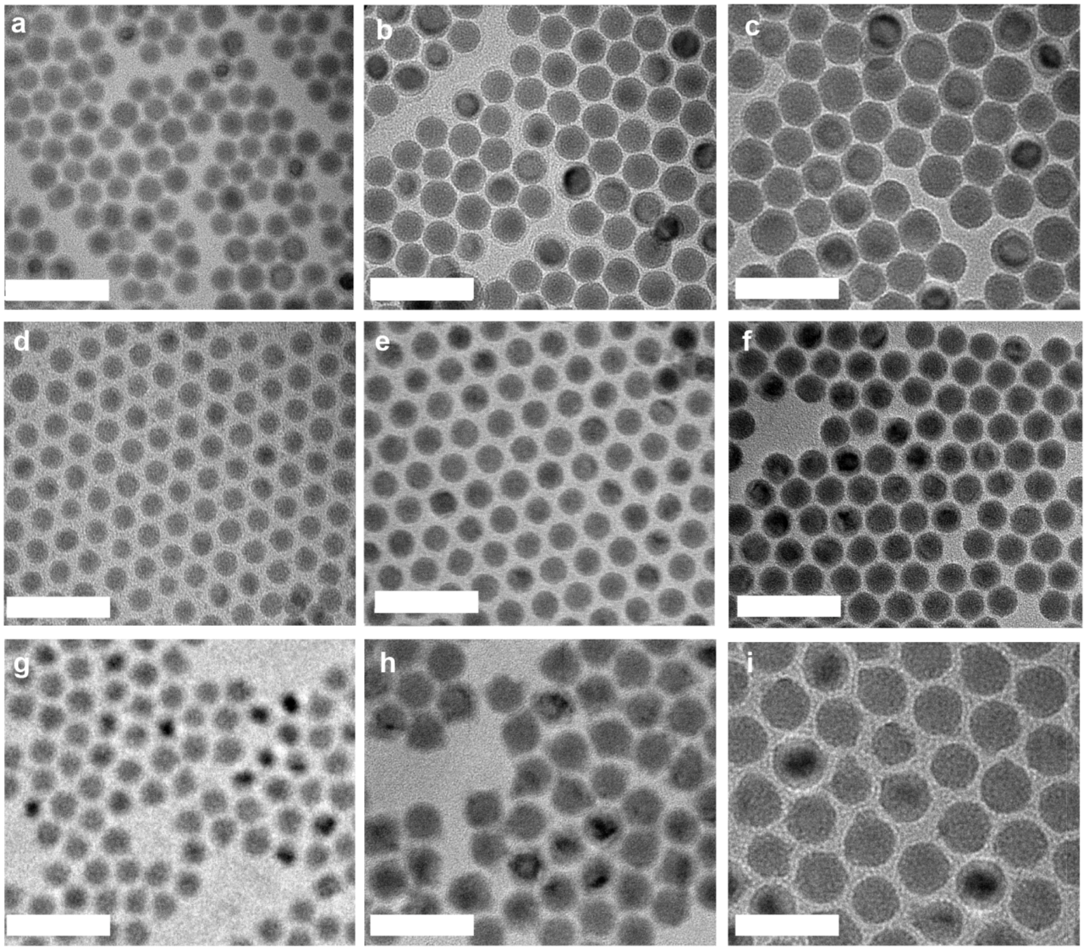

Figure S1. Transmission electron microscopy images of Sn, Bi and In nanocrystals with varying diameters, (a) 11.0 ± 1.2 nm Sn nanocrystals, (b) 15.1 ± 1.0 nm Sn nanocrystals, (c) 17.0 ± 1.1 nm Sn nanocrystals, (d) 9.8 ± 1.0 nm Bi nanocrystals, (e) 13.2 ± 0.7 nm Bi nanocrystals, (f) 14.9 ± 0.7 nm Bi nanocrystals, (g) 11.0 ± 1.1 nm In nanocrystals, (h) 17.0 ± 1.1 nm In nanocrystals, (i) 20.1 ± 1.4 nm nanocrystals (the scale bar in all images is 50 nm).

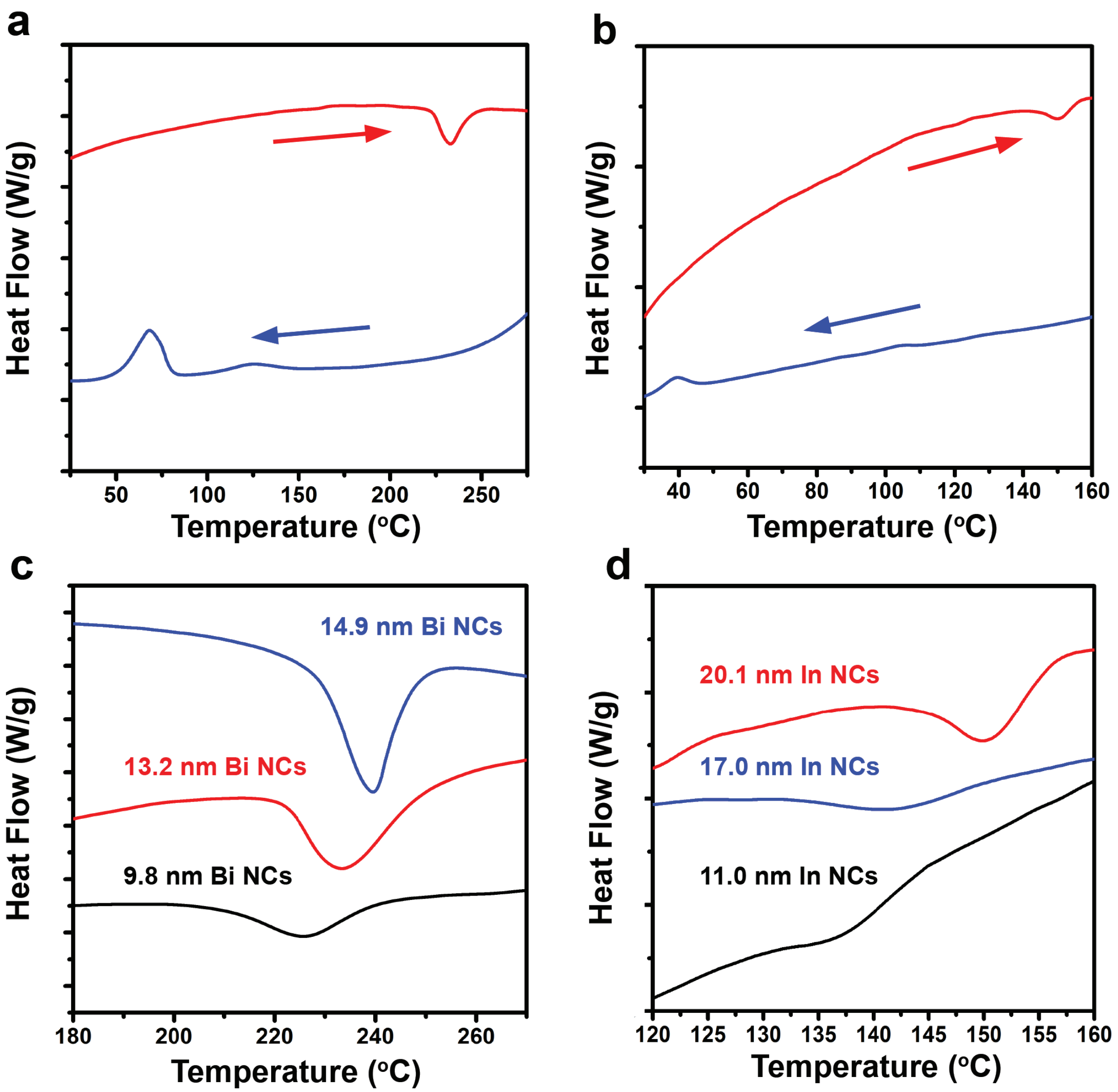

Figure S2. A typical heating (red curve) and cooling (blue curve) cycle during differential scanning calorimetry measurements on (a) 13 nm Bi nanocrystals dispersed in a polyimide resin matrix and (b) 20 nm In nanocrystals dispersed in a polyimide matrix. (c) The endothermic valley of Bi nanocrystals with varying diameters dispersed in a polyimide resin matrix. (d) The endothermic valley of In nanocrystals with varying diameters dispersed in a polyimide resin matrix. As nanocrystal diameter decreases, the melting temperature and melting enthalpy decreases. In addition the full-width at half-maximum of the endothermic melting valley increases.

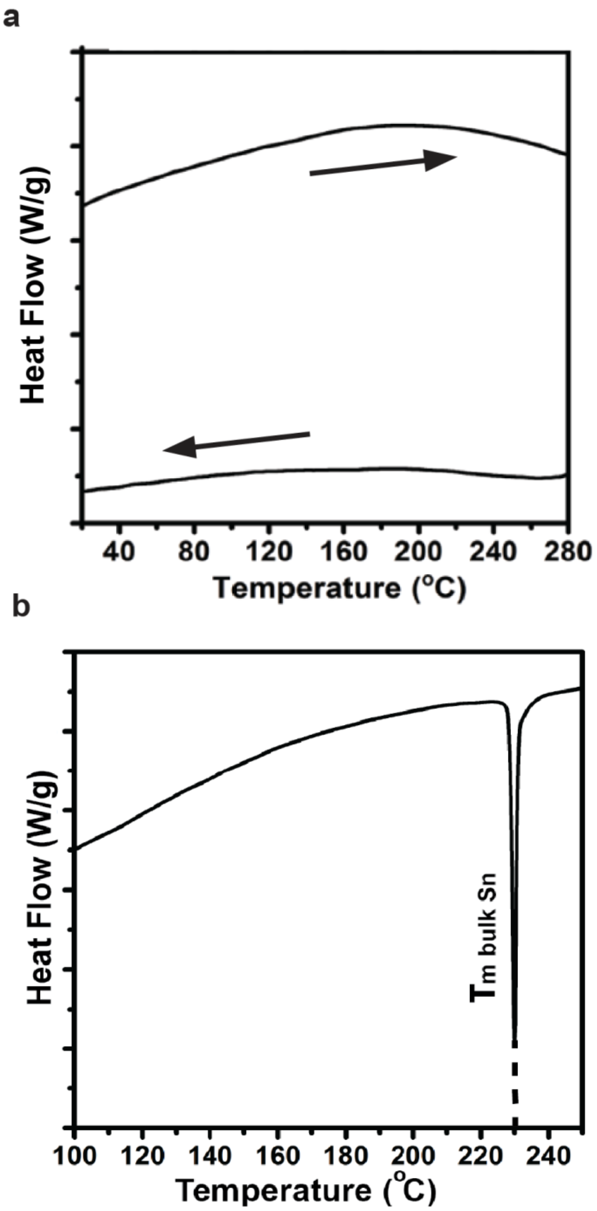

Figure S3. (a) A differential scanning calorimetry measurement on pure polyimide resin. No discernable phase-change features are observed throughout the entire temperature range. (b) A differential scanning calorimetry measurement after several melt-freeze cycles on a sample consisting of only colloidal Sn nanocrystals (i.e. no polyimide resin matrix). Coalescence of the Sn nanocrystals during thermal cycling eventually leads to the bulk melting temperature, 232 °C, being observed. Once this bulk melting temperature is achieved, the mass ratio of the Sn nanocrystal cores to the Sn nanocrystal ligands can be determined by comparing our measured melting enthalpy to the bulk melting enthalpy of tin, 59 J/g.

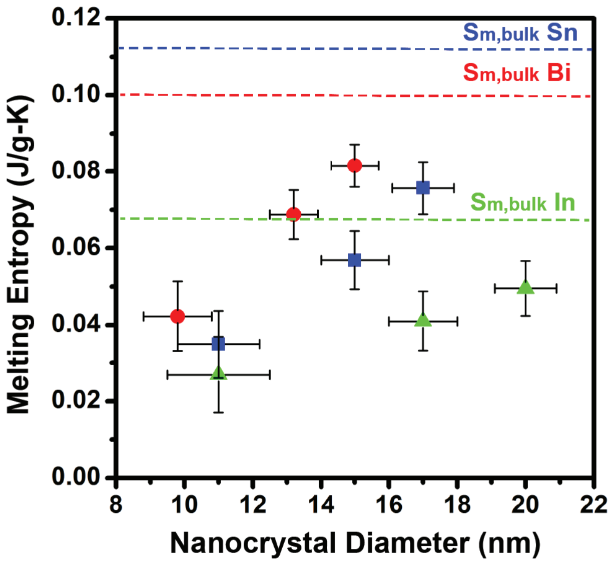

Figure S4. The melting entropy of colloidal In (green triangles), Sn (blue squares), and Bi (red circles) as a function of nanocrystal diameter.

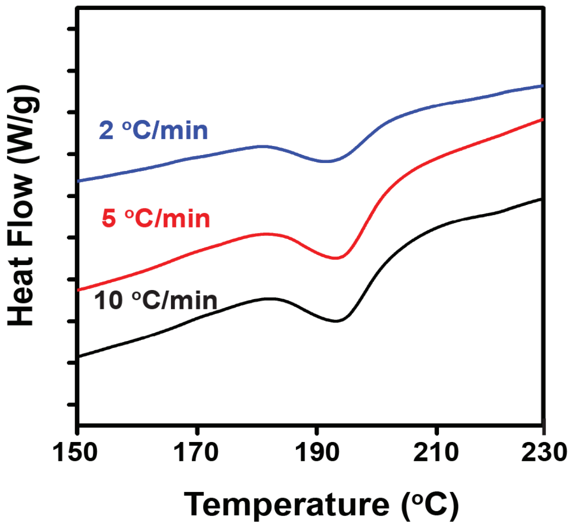

Figure S5. The endothermic valley of 15 nm Sn nanocrystals with different DSC temperature scan rates. No notable changes in the DSC signal are observed over this range of scan rates.

Table S1. Comparison of the full-width at half-maximum (FWHM) of our DSC signals to the DSC signal broadness that can be expected from finite nanocrystal size distribution. The effect of nanocrystal size distribution on DSC signal broadness is calculated using the melting model described by Shi.[1]

Nanocrystal	Diameter (nm)	Standard Deviation of Diameter (nm)	Standard Deviation of Diameter (%)	DSC Signal Broadness due to Nanocrystal Finite Size Distribution (^{o}C)	Measured FWHM of DSC Signal (^{o}C)
Sn	11.0	1.2	10.9	12.2	30
Sn	15.1	1.0	6.7	6.1	24
Sn	17.0	1.1	6.5	3.9	17
Bi	9.8	1.0	10.2	12.4	29
Bi	13.2	0.7	5.3	3.8	18
Bi	14.9	0.7	4.7	2.8	13
In	11.0	1.1	10.0	5.2	36
In	17.0	1.1	6.5	2.3	26
In	20.1	1.4	7.0	2.0	20

References

1. Shi, F. G., Size-dependent thermal vibrations and melting in nanocrystals, *J. Mater. Res.* **9**, 1307-1313 (1994).
